# Human umbilical cord-derived mesenchymal stem cells induce tissue repair and regeneration in collagen-induced arthritis in rats

**Published:** 2020-12-11

**Authors:** Mehak Vohra, Aman Sharma, Rashmi Bagga, Sunil K. Arora

**Affiliations:** ^1^Department of Immunopathology, Post Graduate Institute of Medical Education and Research, Chandigarh, India,; ^2^Department of Internal Medicine (Rheumatology), Post Graduate Institute of Medical Education and Research, Chandigarh, India,; ^3^Department of Obstetrics and Gynaecology Post Graduate Institute of Medical Education and Research, Chandigarh, India

**Keywords:** rheumatoid arthritis, umbilical cord-derived mesenchymal, stem cells, immunomodulation, collagen-induced arthritis, regeneration

## Abstract

The immunosuppressive and anti-inflammatory properties of mesenchymal stem/stromal cells (MSCs) have prompted their therapeutic application in several autoimmune diseases, including rheumatoid arthritis (RA). MSCs derived from bone marrow and adipose tissue has earlier been tried with limited success. However, Wharton’s jelly present in human umbilical cord is discarded after delivery which makes a rich source of MSCs with least ethical issues. The immunomodulatory properties of human umbilical cord-derived MSCs (UC-MSCs) were evaluated *in-vitro* on the mononuclear cells from synovial fluid (SF) and peripheral blood of RA patients. The therapeutic potential of UC-MSCs was checked by transplanting the cells in rats with collagen-induced arthritis (CIA). MSCs isolated from Wharton’s Jelly significantly suppressed the proliferation and activation of lymphocytes from both peripheral blood as well as SF of RA patients, down-modulated the functions of activated CD4+, CD8+ T-cells, suppressed the secretion of pro-inflammatory cytokines, and induced the expansion of T-regulatory cells. Xenotransplantation of UC-MSCs in CIA rats clearly indicated a sustained impact in terms of slowing down the progression of disease activity and reversal of arthritic processes along with triggering of joint tissue repair mechanisms, which could be observed till 6 weeks post-transplantation. The results from the current study suggest that human umbilical cord is a rich source of MSCs for allotransplantation. The UC-MSCs may be used successfully as a cell-based therapeutic option either in isolation or in conjunction with existing therapeutic drugs not only to relieve the joint inflammation but also regenerate the damaged bone and cartilage tissues in arthritis.

**Relevance to Patients:**

The current study highlights the potential use of MSCs as a cell-based therapeutic option for the treatment of inflammatory RA.

## 1. Introduction

Rheumatoid arthritis (RA) is a chronic inflammatory disease that primarily affects the joints, causing articular destruction, associated pain, stiffness, and synovitis [1,2]. In addition to causing a perturbation of both the innate and adaptive immune responses [3,4], RA has been associated with the presence of circulating autoantibodies against self-proteins and rheumatoid factor [5,6]. Current anti-rheumatic therapies primarily target suppression of inflammatory cascade with limited or no success in controlling progression of bone and cartilage destruction [7]. The inflammation-induced bone loss is mediated mainly by increased osteoclast formation and function. Osteoclasts are formed by the fusion of myeloid precursors of monocyte/macrophage lineage in the presence of receptor activator of nuclear factor-kappa B ligand (RANKL) and macrophage-colony-stimulating factor [8]. The pro-inflammatory milieu of the arthritis synovium leads to aberrant differentiation and activation of osteoclasts, resulting in massive bone loss [9]. Therefore, better therapeutic agents are needed which can reverse both inflammatory processes and skeletal damage.

Due to the dual properties of immunomodulation and tissue repair, the mesenchymal stem cells (MSCs) have shown promising results in various autoimmune and degenerative diseases [10]. Initially identified from bone marrow, the MSCs have been isolated from various other sources such as adipose tissue, gingiva, umbilical cord, and many other adult tissues [11]. Several recent studies have shown that bone marrow and adipose tissue-derived MSCs regulate the immune response, including *in vitro* inhibition of T cell proliferation, B cell function, and dendritic cell maturation, though the underlying mechanisms are not fully elucidated [12,13]. In the present study, we used umbilical cord tissue-derived MSCs (UC-MSCs), mainly because the cord is a discard material after delivery of the child and thus raises least ethical issues; besides, the MSCs are relatively easier to isolate from the Wharton’s jelly, where these are present in abundance. While, aspirating bone marrow and collecting adipose tissue is a painful and invasive procedure. In addition, the number and the differentiating potential of MSCs isolated from these sources decrease with age [14]. Numerous studies have reported that MSCs from various sources have immunomodulatory effects on RA [15-18], but effects on the inhibition of joint destruction in rats upon external administration of cord matrix derived MSCs still remain unknown. In our study, the UC-MSCs showed strong anti-inflammatory effects on lymphocytes isolated from the SF and peripheral blood of RA patients *ex vivo* while inducing a sustained regeneration of inflammation-induced degenerated bone and cartilage along with reduced differentiation of bone-resorbing osteoclasts in rats with collagen-induced arthritis (CIA), indicating a possibility of using UC-MSCs as a cell-based therapeutic option in RA.

## 2. Materials and Methods

### 2.1. Isolation, culture, and identification of Wharton’s Jelly MSC

This study was approved by the ethical committee of Postgraduate Institute of Medical Education and Research, Chandigarh, India, with reference no INT/IEC/2019/000016. Fresh Human Umbilical Cord samples (*n*=12) were collected from normal healthy females delivering babies in the “Clean Labor Room” of the Department of Obstetrics and Gynaecology, Postgraduate Institute of Medical Education and Research, Chandigarh, India. The cord transported to laboratory under sterile and cold conditions was washed with Phosphate Buffered Saline (PBS). The Wharton’s Jelly, after removal of cord arteries and veins, was cut into small explants of approximately 1 mm^3^ size and left undisturbed in sterile Petri plates containing α-Minimum Essential Medium (MEM) supplemented with 10% fetal bovine serum (FBS), L-Glutamine, and antibiotics in an incubator at 37°C with 5% CO_2_ for 10-12 days. The MSCs radiating out of the tissue explants were passaged on confluence every 5-7 days. The cells were allowed to grow till passage 4-5. The P4 generation cells were retrieved by trypsinization (treating the confluent adherent cells with 0.25% trypsin containing 1 mM ethylenediaminetetraacetic acid for 3-4 min) and stained with fluorochrome-conjugated monoclonal antibodies to cell surface markers- anti-HLA-DR-Allophycocyanin (APC), anti-CD34-BV421, anti-CD45-Fluorescein isothiocyanate (FITC), anti-CD105-PE, anti-CD90-APC, and anti-CD73-BV605 (BD Biosciences, USA) for phenotypic characterization in a multi-laser flow-cytometer (fluorescence-activated cell sorting-[FACS] Aria, BD Biosciences, USA).

The cells were checked for their differentiation potential into adipogenic, osteogenic, and chondrogenic lineages, using a specific induction medium (HiMedia, India) as per manufacturer’s instructions. Briefly, the UC-MSCs were maintained in complete a-MEM till 80-85% confluency and then specific induction media was used to initiate differentiation. The UC-MSCs were maintained for 21 days in an induction medium which was replaced every 3^rd^ day. After completion of induction time (21 days), cells were stained with Oil Red O for lipid droplets, Alizarin Red S for calcium deposits and Alcian Blue stain for aggrecan, respectively.

### 2.2. Co-culture with T lymphocytes from synovial fluid and peripheral blood of RA patients

Confirmed RA patients on disease-modifying anti-rheumatic drugs (DMARDs) and still showing disease activity score (DAS28) 3.2 and above were recruited in the study. The peripheral blood and SF samples of patients (*n*=15) were collected in a paired manner after informed consent. Mononuclear cells (MNCs) were isolated from heparinized blood and SF by Ficoll-Hypaque density gradient centrifugation (HiMedia, Mumbai, India) and labeled with carboxyfluorescein succinimidyl ester (CFSE). For labeling, 5-6*10^6^ cells were suspended in pre-warmed PBS containing 0.1% bovine serum albumin and 5mM CFSE. After incubating cells at 37°C for 10 min in the dark, cells were washed 2-3 times with cold plain RPMI 1640.

For co-culture, 1*10^4^ UC-MSCs were seeded per well in complete RPMI 1640 medium (containing 10% FBS, 1% Penicillin-Streptomycin, and 2 mM L-Glutamine) along with 1*10^5^ MNCs from SF or peripheral blood (1:10 ratio) in the presence of 4 ug/ml of phytohaemagglutinin (PHA) for 72 h [19]. The CFSE dye dilution and expression of activation markers (CD25, CD38, and HLA-DR) on MNCs were evaluated by flow cytometry using FACS Diva™ Software (BD, Biosciences).

### 2.3. Th-17 and Treg cell evaluation

After 5 days of co-culture setup, the cells were harvested from each of the wells and the phenotypes of the different T-cell subsets were assessed by FACS analysis using a set of cell surface markers, intracellular transcription factors, and secreted cytokines. Surface staining was carried out for 15 min at RT with monoclonal antibody conjugates anti-CD3-PerCpCy5.5, anti-CD4-FITC. For intracellular staining, cells were permeablized and stained with anti-interferon (IFN)**-**γ**-**PE, anti-interleukin (IL)-17-Alexa Fluor 647, anti-IL-4-BV421, anti-IL-2-FITC, and anti-FoxP3-PE. The cells were acquired in a multi-laser flow cytometer (BD FACS ARIA II; BD Bioscience, USA) for analysis.

### 2.4. Cytotoxic T-cells (CD8+T-cells)

The CD8+ cells (CD3+CD4^neg^ subset) were analyzed for production of granzyme B, perforin, IFN-γ, and IL-17 by flow cytometry. The MNCs were stained for cell surface markers CD3, CD4 (as above) and subsequently permeabilized and fixed. Intracellular staining with monoclonal antibodies for granzyme B and perforin was carried out and cells were analyzed on BD FACS ARIA II flow cytometer (BD Bioscience, USA).

### 2.5. Secreted cytokine analysis

Quantification of various cytokines, namely, IL-1β, IL-6, IL-8, IL-10, tumor necrosis factor (TNF)-α, and IL-12p70 was performed from the culture supernatant of all the three experimental groups using Cytokine Bead Array human inflammatory cytokine kit (BD Bioscience, USA) analyzed using FCAP Array software (BD Biosciences, USA). Human transforming growth factor (TGF-β) was assessed using ELISA Kit (RayBio Human TGF-β ELISA Kit) according to manufacturer’s instructions.

### 2.6. Induction of CIA in rats

For CIA induction, young (180-200 g) female Wistar Rats were immunized intradermally at the base of the tail with 100 mg chicken collagen type II (CII; Sigma, USA) emulsified in complete Freund’s adjuvant (CFA; Sigma, USA), followed by an injection of bacterial lipopolysaccharide (1 mg/ml LPS) after 2 weeks and a booster dose on day 15^th^ with 100 μg CII in incomplete adjuvant [20]. Passage four (P-4) UC-MSCs were infused (2×10^6^ cells per rat on alternate days 16 and 18) into a group (*n*=5) of CIA rats intraperitoneally, while in the untreated group, the CIA-rats received only PBS. All the rats were monitored for short-term effects (2 weeks) and long-term effects (6 weeks). Control rats were not immunized with CII and not treated with UC-MSCs. All animal experiments were approved by Institutional Animal Ethics Committee with reference no. 105/85/82/IAEC/530.

Rats were evaluated twice weekly for signs of arthritis based on paw swelling and arthritis scores. Clinical arthritis in CIA rats post-MSC treatment was evaluated in a blinded manner by a trained rheumatologist assigning “arthritis scores” as no swelling=0; only wrist swelling=1; swelling in wrist and paw=2; swelling in wrist, paw, and digits=3; and whole paw swollen=4. Paw and tibiotarsal joint thickness was evaluated using digital Vernier Caliper and paw volume measurement using a water-based plethysmometer.

### 2.7. Histological analysis

For histopathological examination, after 2 weeks and 6 weeks post stem cell treatment animals were sacrificed, the limbs of untreated and treated CIA rats fixed in 10% paraformaldehyde were decalcified and 5-μm sections of paraffin-embedded tissue blocks were stained with hematoxylin and eosin to examine the synovial inflammation, bone erosion, and cartilage damage [21]. Synovial inflammation was scored as 0 for no inflammatory cells; 1 for slight thickening of the epithelial lining with few infiltrating cells in the sub-lining layer; 2 for moderate thickening of the lining with a moderate number of infiltrating cells in the sub-lining layer; 3 for extensive thickening of the lining with a moderate number of infiltrating cells and the presence of inflammatory cells in the synovial space; and 4 for a substantial influx of inflammatory cells into the synovium. Bone erosion was also scored as 0 for no erosion; 1 for small areas of resorption not readily apparent in the trabecular or cortical bone; 2 for numerous areas of resorption, readily apparent in the trabecular or cortical bone at low magnification; 3 for obvious resorption of the trabecular and cortical bone without full-thickness defects in the cortex, but with the loss of some trabecular bone; 4 for full-thickness defects in the cortical bone and marked loss of trabecular bone with no distortion of the remaining cortical surface; and 5 for full-thickness defects in the cortical and trabecular bone with distortion of the remaining cortical surface. Cartilage damage was scored as: 0 for no destruction; 1 for minimal erosion; 2 for slight to moderate erosion in a limited area; 3 for extensive erosion; and 4 for general destruction. For the control rats (not immunized, not treated), a score of 0 was given for all three parameters. Complete autopsy and histopathological examination of liver, kidney, and spleen of all the untreated as well as MSC-treated CIA rats were conducted to evaluate any adverse impact of MSC-treatment.

### 2.8. X-ray analysis

After 2 weeks and 6 weeks post stem cell treatment, before sacrificing, the rats were X-ray radiographed for any radiological changes (SKANMOBILE, Skanray Technologies Ltd.). Radiologic scores were assigned according to the criteria: 0 for no radiologic changes; 1 for mild changes with tissue swelling and edema; 2 for moderate changes with joint erosion and disfiguration; and 3 for severe changes with bone erosion and osteophyte formation. For the control rats (not immunized, not treated), a score of 0 was given because no radiological changes were seen.

### 2.9. Gene expression profiling

Total RNA was isolated using a total RNA isolation reagent (Sigma-Aldrich, USA) from tibiotarsal joint tissue of control, untreated, and MSC-treated CIA rats. Complementary DNA was generated by reverse transcription reaction using RevertAid First Strand cDNA synthesis Kit (Thermo Scientific). Real-time quantitative polymerase chain reaction (PCR) was performed using SYBR Green Master Mix (Qiagen, Germany) in a LC480 (Roche, Germany) real-time PCR machine. Real-time PCR was performed with an initial denaturation step of 10 min at 95°C, followed by 40 cycles of 15 s at 95°C and 30 s at an annealing temperature of 63°C and 45 s at 72°C. The relative quantitative expression levels of the genes related to the bone erosion (TRAP, Cathepsin K, RANK, RANKL, NFATc1, Osteoprotegerin [OPG]), and cartilage (MMP3, MMP9) damage were evaluated after normalizing to levels of GAPDH and b-Actin as endogenous controls and compared to a normal control rat sample as a reference for calculation of ∆∆ Ct values (Table 1).

**Table 1:** Real-time polymerase chain reaction primer pairs.

Gene name	Forward primer (5’-3’)	Reverse primer (5’-3’)
Cathepsin K	TCCTCAACAGTGCAAGCGAA	CCAGCGTCTATCAGCACAGA
TRAP	GTGCATGACGCCAATGACAAG	TTTCCAGCCAGCACGTACCA
MMP9	TCGAAGGCGACCTCAAGTG	TTCGGTGTAGCTTTGGATCCA
MMP3	TTTGGCCGTCTCTTCCATCC	GCATCGATCTTCTGGACGGT
RANKL	TGGGCCAAGATCTCTAACATGA	TCATGATGCCTGAAGCAAATG
RANK	GCCCAGTCTCATCGTTCTG C	GCAAGCATCATTGACCCAATTC
OPG	GTGTGTCCCTTGCCCTGACTAC	GTTTCACGGTCTGCAGTTCCTT
NFATc1	CCCTAGCGTCACCTCAACTC	TGGTGAAGGCCCAGATAGGA
GAPDH	TCAAGGGCATCCTGGGCTAC	CGTCAAGGTGGAGGAGTGG
β-ACTIN	CCCATCTATGAGGGTTACGC	TTTAATGTCACGCACGATTTC

#### 2.9.1. Tissue cytokine profile

Concentrations of TNF-α and IL-10 in tibiotarsal joint tissue homogenates and corresponding serum samples were estimated using rat TNF-α and IL-10 ELISA kits (Elabscience, India) according to the manufacturer’s instructions.

### 2.10. Statistical analysis

Results are expressed as mean±standard error of the mean (SEM). Statistical significance of difference was determined using the Mann–Whitney U test for comparison between two unpaired groups, Kruskal–Wallis test for comparison between different groups. GraphPad Prism 6 software was used for all statistical analyses. *P*<0.05 was considered statistically significant.

## 3. Results

### 3.1. Human UC-MSCs exhibit multi-lineage differentiation and immunosuppressive potential

**Figure 1:**
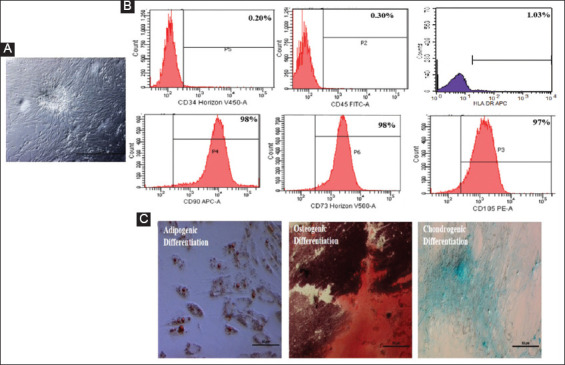
Phenotypic and functional characterization of umbilical cord-derived mesenchymal stem cells (UC-MSCs). (**A**) Representative photomicrograph of cultured UC-MSCs, (**B**) flow cytometric analysis indicating MSCs negative for cell surface markers: CD34, CD45, and HLA-DR but positive for CD105, CD90, and CD73, (**C**) microscopic assessment of MSC’s differentiation potential after 21 days under specific induction medium. Differentiated adipocytes containing lipid droplets stained with oil red o stain, differentiated osteocytes containing calcium deposits stained with alizarin red S, and differentiated chondrocytes containing aggrecan stained with Alcian Blue. Magnification ×40, Scale bar 50 mm.

**Figure 2:**
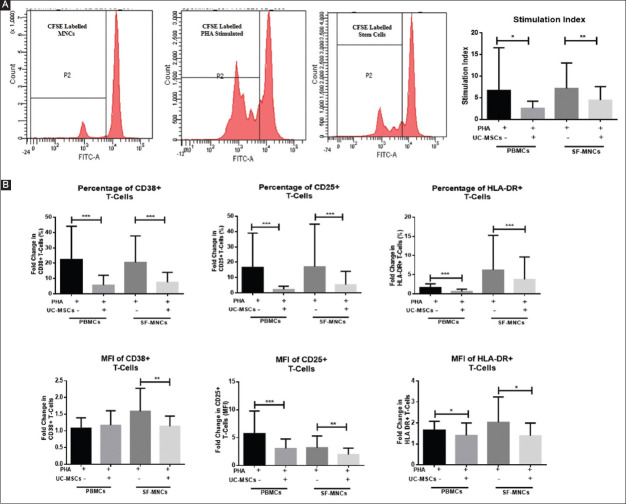
Immunosuppressive effect of umbilical cord-derived mesenchymal stem cells (UC-MSCs) on lymphocyte proliferation and activation status. (**A**) Histograms showing comparative average stimulation indices of carboxyfluorescein succinimidyl ester-stained PHA stimulated peripheral blood mononuclear cell (PBMCs) and SF-MNCs of rheumatoid arthritis subjects with or without co-cultured mesenchymal stem cells. (**B**) Fold change in percentage and MFI of T-cells expressing activation markers – CD38, CD25, HLA-DR on PBMCs, and SF-MNCs in different study groups over unstimulated control. Results represent Mean±standard error of the mean. *P<0.05, **P<0.005, ***P<0.0005. MFI: Mean fluorescence intensity; PHA: Phytohaemagglutinin.

MSC isolated from the Wharton’s jelly of the umbilical cord tissue using explant method (UC-MSCs) exhibited fibroblast-like morphology in culture (Figure 1A) and were strongly positive for surface expression of MSC-specific markers CD90, CD105, CD73, and negative for the hematopoietic markers CD34 and CD45 (Figure 1B). In lineage-specific differentiation conditions, the UC-MSCs formed mineralized nodules in the presence of osteogenic factors as confirmed by Alizarin Red S staining; formed intracellular oil globules on adipogenic induction as confirmed by Oil Red O staining and differentiated into chondrocytes as indicated by expression of aggrecan protein under chondrogenic differentiation conditions, which turned blue on staining with Alcian Blue (Figure 1C). The UC-MSCs, significantly inhibited the proliferation of PHA stimulated MNCs from peripheral blood (SI±SEM: 2.5±0.4 vs. 6.6±2.74) and SF (SI±SEM: 4.5±0.8 vs. 7.15±1.5) of RA patients in co-culture experiments (Figure 2A). The UC-MSCs also significantly suppressed the expression of activation markers CD38, CD25, and HLA-DR both on peripheral blood mononuclear cells (PBMCs) and SF-MNCs from RA patients in these co-culture settings (Figure 2B).

### 3.2. UC-MSCs skew Th1/Th2/Th17 cytokine balance

**Figure 3:**
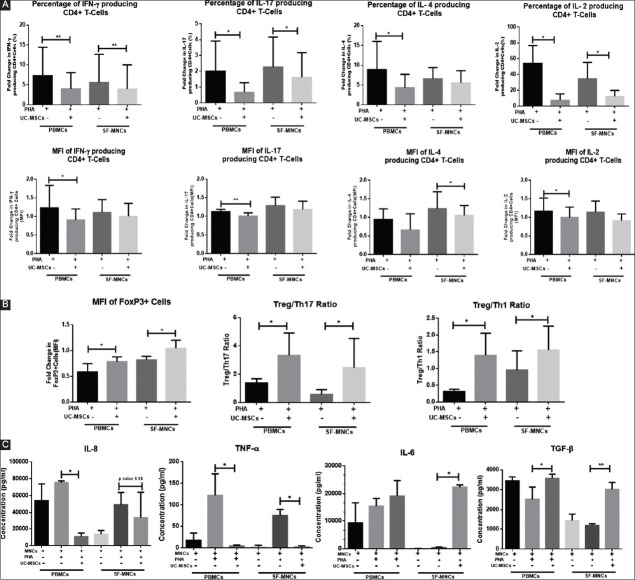
Immunosuppressive effect of umbilical cord-derived mesenchymal stem cells on cytokine production and T-regulatory cell induction. (**A**) Fold change in percentage and mean fluorescence intensity (MFI) of CD4+ T-cells from peripheral blood mononuclear cells (PBMCs) and synovial fluid mononuclear cells (SF-MNCs) of rheumatoid arthritis patients producing intracellular cytokines- interferon-g, interleukin (IL)-2, IL-4, and IL-17 in different experimental groups. (**B**) Fold change in MFI of T-regulatory cells in various experimental groups of PBMCs and SF-MNCs and an increased Treg/Th17, Treg/Th1 ratio. (**C**) Cytokine (IL-8, IL-6, TNF-α, and transforming growth factor-b) levels in culture supernatant of PBMCs and SF-MNCs from rheumatoid arthritis patient in different experimental groups of MNCs alone, phytohaemagglutinin (PHA) stimulated MNCs, and stem cell co-cultured with PHA stimulated MNCs. Results represent mean±standard error of the mean. *P<0.05, **P<0.005.

In the co-culture set-up, the UC-MSCs significantly down modulated the production of cytokines: IFN-ϒ (Mean±SEM: 2.86±0.85 and 7.29±2.51); IL-17 (Mean±SEM, 0.66±0.18 and 2.00±0.63); IL-4 (Mean±SEM, 4.31±1.2 and 12.06±4.0); and IL-2 (Mean±SEM, 7.53±3.59 and 45.23±12.55) by CD4 cells from PBMCs as well as SF-MNCs (Mean±SEM, IFN-ϒ: 6.18±2.87 and 8.75±3.81, IL-17: 1.62±0.51 and 2.26±0.63, IL-4: 5.54±1.26 and 6.56±1.16, and IL-2: 12.47±3.35 and 34.57±9.30) as seen by intracellular cytokine staining (Figure 3A). In addition, production of pro-inflammatory cytokines such as TNF-α and IL-8 was decreased while the level of IL-6 and TGF-β was increased in the culture supernatants of stem cell-treated MNCs from peripheral blood and SF *ex vivo*. Thereby, these results indicate that MSCs skew the environment from pro-inflammatory type to anti-inflammatory type (Figure 3C).

### 3.3. UC-MSCs induce T-regulatory cells expansion

Multicolor FACS analysis of 5 days co-culture set-up of UC-MSC with PHA-stimulated MNCs from peripheral blood and SF-MNCs revealed that the percentage of T-regulatory (Treg) cell (CD3+ CD4+ CD25+ FoxP3+) component was significantly increased (Mean±SEM 3.09±0.54 and SF-MNCs Mean±SEM, 2.15±1.61) in comparison to cultures without UC-MSCs (Mean±SEM 2.5±0.21 and 1.66±0.37). Besides, there was a significant quantitative increase in the expression of FoxP3 in MNCs from peripheral blood (Mean±SEM, 0.78±0.03) and SF-MNCs (Mean±SEM, 1.04±0.06) when co-cultured with UC-MSCs as compared to without UC-MSCs (Mean±SEM: 0.58±0.06 and 0.82±0.02, respectively) (Figure 3B), suggesting the expansion of activated Treg cells in the presence of UC-MSCs.

### 3.4. UC-MSCs inhibit the production of cytotoxic granules and pro-inflammatory cytokine by CD8+ T-cells

**Figure 4:**
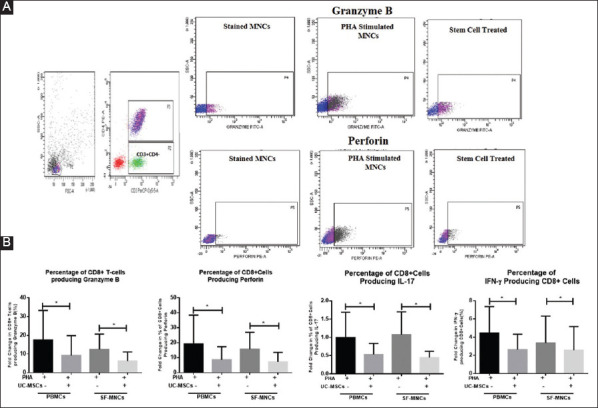
Mesenchymal stem cells decrease the percentage and quantitative production of granzyme B, perforin, and pro-inflammatory cytokines by CD8+ T-cells (in terms of fold change in percentage and mean fluorescence intensity) both in case of peripheral blood mononuclear cell and synovial fluid mononuclear cells from rheumatoid arthritis patients. (**A**) Representative flow cytograms; (**B**) histograms showing comparative data. Results represent Mean±standard error of the mean. *P<0.05.

Granzyme B and perforin production by CD8+ T cells play a significant role in the RA disease pathogenesis. Through multicolor FACS Analysis, we investigated the effect of stem cells on the production of lytic enzymes such as granzyme B and perforin produced by CD8+ T-cells in peripheral blood and SF of RA patients. We found that stem cells significantly decreased the frequency of CD8+ T-cells producing granzyme B (Mean±SEM, 9.21±4.34) and perforin (Mean±SEM, 8.73±3.52) as compared to mitogen (PHA) stimulated lymphocytes in the absence of MSCs (granzyme B Mean±SEM, 17.44±6.40 and perforin Mean±SEM, 19.21±7.8). A similar trend of decrease was observed in SF-MNCs when cultured with UC-MSCs. In addition to IFN-γ and IL-17 production by CD4+ T-cells, we also analyzed the production of these cytokines by CD8+ T-Cells and found a decrease in percentage and mean fluorescence intensity in PBMCs and SF-MNCs after stem cell treatment. Thereby, indicating immunosuppressive effect of UC-MSCs on both CD4+ and CD8+ T-cell effector functions (Figure 4).

### 3.5. Anti-arthritis effects of UC-MSCs in CIA rats

#### 3.5.1. Effect on paw thickness, paw volume, and arthritis scoring of CIA rats

**Figure 5:**
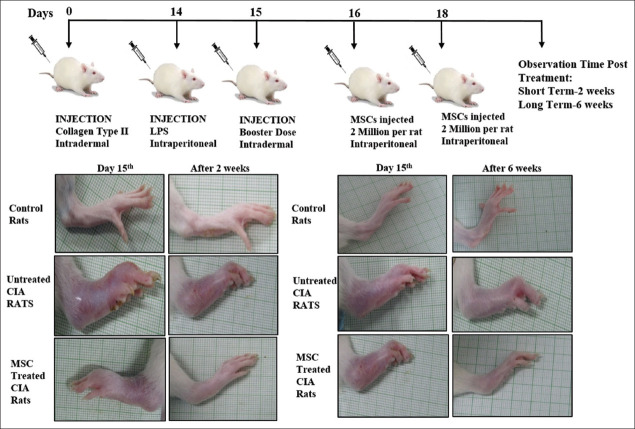
Induction of collagen-induced arthritis (CIA) in female Wistar rats and umbilical cord-derived mesenchymal stem cells treatment regime followed. Representative images of control, untreated CIA, and mesenchymal stem cell-treated CIA rats showing the clinical signs of the disease: Redness, paw, and tibiotarsal joint swelling both before and after 2 weeks and 6 weeks of stem cell treatment. Magnification ×1.

**Figure 6:**
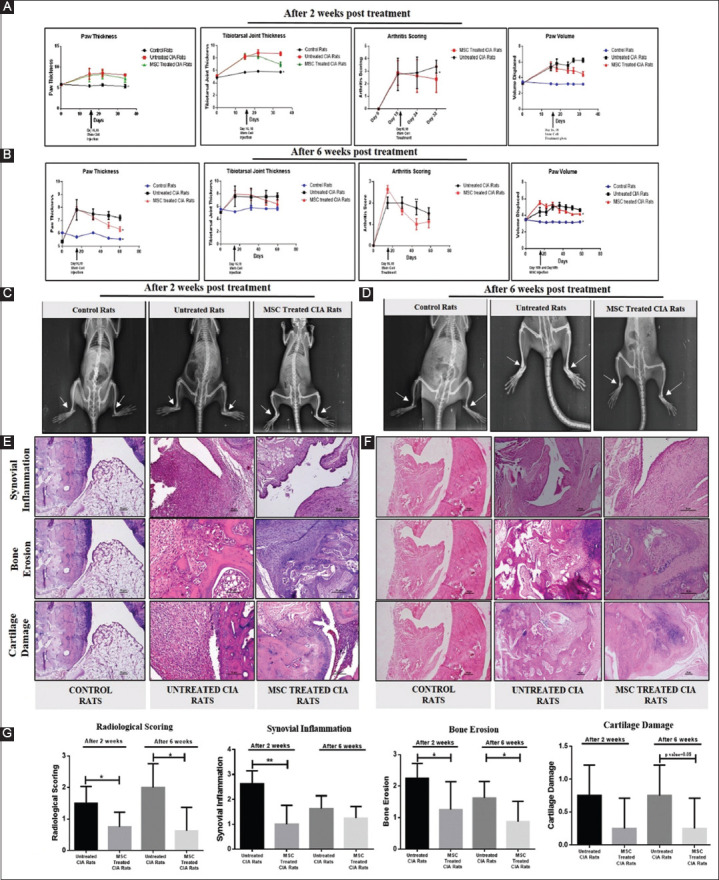
Transplantation of umbilical cord-derived mesenchymal stem cells alleviated symptoms in CIA rats in a sustained manner. (**A and B**) Line diagrams showing measurement of hind paw and tibiotarsal joint thickness by Digital Vernier Caliper, paw volume by plethysmometer, and arthritis scoring at different time points (2 weeks and 6 weeks post-treatment) in different groups. Group 1-control rats (not immunized), Group 2-untreated CIA rats, Group 3-mesenchymal stem cell-treated CIA rats (*n*=4 in each group). (**C and D**) Representative radiographs (white arrowhead) of bone erosion in hind paws from different animal groups post 2 weeks and 6 weeks of treatment. (**E and F**) The 0.5-micron paraffin-embedded tissue sections were stained with hematoxylin and eosin to study the degree of synovitis and bone-cartilage erosions in different animal groups. (**G**) Changes in radiological and histological scoring for each animal group. Results represent Mean±standard error of the mean. *P<0.05, **P<0.005. Magnification ×20 and ×10, Scale bar 50 mm.

CIA is the most studied model for RA. Many similarities with human RA make CIA an ideal animal model for conducting studies to assess the therapeutic potential of various agents for the treatment of RA. The visible signs of arthritis-like paw swelling, increased arthritis index and redness were observed in CIA rats, usually within 12-14 days from primary immunization (Figure 5). Our results exhibited that administration of UC-MSCs at a dose of 2 million cells per rat on days 16^th^ and 18^th^ significantly decreased the swelling in paw (day 32, 7.18±0.31) and tibiotarsal joint (day 32, 7.00±0.37) in treated CIA rats as compared to untreated CIA rats (paw thickness, day 32, 8.03±0.10 and tibiotarsal joint thickness, day 32, 8.68±0.29) post 2 weeks of treatment. In addition, we also found significantly decreased arthritis index and paw volume of UC-MSC-treated CIA rats (day 32, arthritic index 2.37±0.37, paw volume 4.41±0.28) as compared to the untreated CIA rats (day 32, arthritic index 3.37±0.18, paw volume 6.22±0.24). Further, the UC-MSC treatment mediated slowing down of the progression of CIA in rats was found to be sustained even till 6 weeks (Day 60^th^) of post-treatment (Figure 6A and B).

#### 3.5.2. Sustained anti-arthritis role of UC-MSCs was confirmed by radiological and histological evaluation

After 2- and 6-week post-treatment, X-ray radiographs were taken to further assess the therapeutic effects of UC-MSCs. Radiological scorings were done using X-ray images, based on parameters such as soft tissue swelling, cartilage and bone destruction, joint narrowing, and bone loss. Rats without arthritis (control rats) showed normal soft tissue and joint structure. CIA rats showed severe swelling of the soft tissues along with bone erosion and a narrowed joint space (Figure 6C and D; indicated by white arrows). In the UC-MSC-treated CIA rats, the soft tissue of each immunized paw was observed to be only slightly swollen with lesser joint destruction in comparison to untreated CIA rats. The radiological scoring revealed a sustained decrease with stem cell treatment at 2 weeks (day 32, Mean±SEM, 0.75±0.16), further maintained till 6 weeks also (day 60, Mean±SEM, 0.62±0.26) as compared to untreated CIA rats, thereby indicating that the UC-MSC treatment induced a sustained reduction in the bone and cartilage damage in CIA Rats (Figure 6G). The histopathological investigation of UC-MSC-treated CIA rats sacrificed after 2 or 6 weeks of treatment revealed near-normal structures of the joint cavities with unremarkable synovial hyperplasia and markedly reduced infiltration of inflammatory cells in comparison to histological features of the distortion of the normal structure of joints along with thickening of synovial membrane and infiltration of a large number of lymphocytes and polymorphonuclear cells in paws of untreated CIA rats. The bone erosion caused by giant osteoclastic cells and cartilage damage caused due to the production of matrix metalloproteinases in MSC-treated CIA rats was significantly lesser than those of the untreated CIA rats. Moreover, these changes were observable till 6 weeks of observation post-treatment (Figure 6E and F).

#### 3.5.3. UC-MSCs decrease osteoclastogenesis in arthritis rat by reducing the gene expression of osteoclast precursors

**Figure 7:**
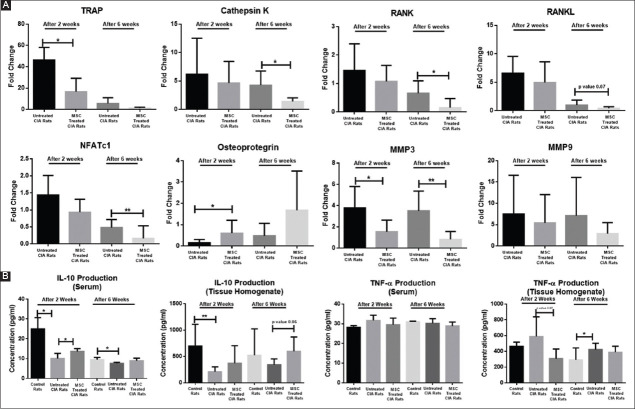
Prolonged effect of umbilical cord-derived mesenchymal stem cells on osteoclastogenesis and cytokine production. (**A**) Differential gene expression analysis of genes related to bone and cartilage damage in synovial tissue of untreated CIA and mesenchymal stem cell (MSC)-treated CIA after 2 and 6 weeks of treatment. (**B**) Decrease in pro-inflammatory (TNF-α) and increase in anti-inflammatory cytokine (IL-10) levels in serum and tibiotarsal joint homogenate of control, untreated CIA and MSC-treated CIA rats (post 2 and 6 weeks treatment). Results represent Mean±standard error of the mean. *P<0.05, **P<0.005.

The histological scoring revealed reduced areas of bone resorption after treatment, which is most likely due to the reduced number of osteoclastic cells that cause bone damage. Hence, it may be highlighted that MSC-treatment protected bone damage till 6 weeks of observation, partly through suppressing the number and functions of osteoclastic cells in CIA rats as evident from our histological investigation of affected joints of UC-MSC-treated compared to untreated CIA rats. To further validate these results, we selected the marker genes associated with osteoclast differentiation and activation at the transcript level and studied their quantitative expression profile in healthy control, untreated CIA, and stem cell-treated CIA rats. The relative gene expression data by real-time PCR showed that treatment with UC-MSCs downregulated the expression of both osteoclast-inducing genes (e.g., RANKL, RANK, and NFATc1) as well as osteoclast-specific genes (TRAP, Cathepsin K), in the tibiotarsal joints of the CIA rats in comparison to untreated rats (Figure 7A). Further, the expression of OPG gene, a RANKL decoy receptor, was found to be significantly increased in UC-MSC-treated CIA rats. The MSC treatment also suppressed the expression of matrix metalloproteinases (MMPs), which are known to be involved in cartilage damage. The imbalance between pro-inflammatory and anti-inflammatory cytokines is known to contribute significantly to the pathogenesis and development of RA. Interestingly, the level of TNF-α was increased in untreated CIA rats, while IL-10 was low. However, these trends were found to be reversed in the MSC-treated CIA rats, both in the serum and tissue homogenate of the synovial tissue (ST), as estimated after 2 weeks and 6 weeks of treatment (Figure 7B). Thus, MSCs not only suppressed the formation of osteoclasts but also down-modulated the immune and inflammatory responses that indirectly attenuated the osteoclasts, eventually contributing to bone protection in CIA.

### 3.6. MSC treatment had no adverse effect on vital organs – liver, kidney, and spleen

**Figure 8:**
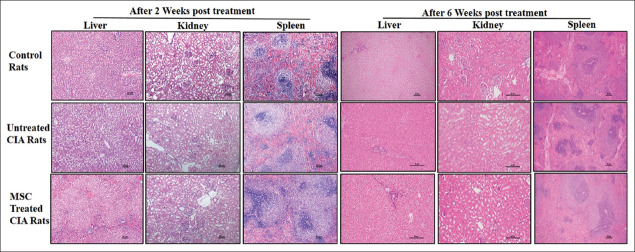
No obvious side effects of umbilical cord-derived mesenchymal stem cells transplantation. Hematoxylin and eosin staining of – liver, spleen, and kidney tissues from control, untreated CIA and mesenchymal stem cell-treated CIA rats post 2 and 6 weeks of treatment. Magnification ×20 and ×10, Scale bar 50 mm.

Histopathological examination of liver, kidney, and spleen tissues of UC-MSC-treated CIA rats revealed no histological changes even till 6 weeks post-treatment. In the case of kidney, no tubular damage or inflammation was observed. No cellular damage or infiltration of immune cells was observed in livers of any of the UC-MSC-treated CIA rats and no inflammatory cells were seen in the white and red pulp areas of spleen (Figure 8). Similar results were obtained post 2 weeks of treatment (Figure 8).

## 4. Discussion

RA in humans is a systemic chronic autoimmune disease affecting multiple organs in the body, causing predominantly inflammatory synovitis and structural damage to bone and cartilage [22]. At present, the inflammatory synovitis is managed by conventional DMARDs, which can suppress the inflammation, but for protection against structural damage, the patient would require complete abrogation of inflammation along with regeneration of damaged bone and cartilage in affected joints. Therefore, some novel agents having the capability to manage both inflammatory and skeletal components of the disease would be an appropriate alternative treatment modality in such conditions. MSCs have emerged as a cell-based therapeutic option in various autoimmune and degenerative diseases [23-25]. The immunosuppressive capabilities of MSCs against various autoimmune reactions are better than those of any other immunosuppressive cell described until now [20,26]. Bartholomew *et al*. showed for the 1^st^ time that the MSCs not only escaped recognition by alloreactive T cells when added to mixed lymphocyte reactions *in vitro*, but they suppressed the T-cell proliferation also [27]. Studies conducted by Gonzalez *et al*. have shown that MSCs suppress functions of immune cells directly by releasing TGF-β and IL-10 or indirectly by induction of regulatory T (Treg) cells, both *in vitro* and *in vivo* [28]. The immunomodulatory potential of MSCs isolated from various sources has earlier been reported in various chronic inflammatory conditions, including RA, but all of these sources have one or the other limitations as far as their anticipated use in humans is concerned. We isolated the MSC from the human umbilical cord matrix (UC-MSCs) in the current study. The Wharton’s jelly in the human umbilical cord is a very rich source of MSCs besides being a discard material after delivery of the baby and thus invites the least ethical concerns. It is much easier to retrieve a large number of pure population of these cells from the cord matrix as compared to cord blood. A study conducted by Mustapha *et al*. has shown that the yield of MSCs recovered from UCB is too low to be considered as a reliable source for further use and confirmed that umbilical cord matrix is a rich source of MSC [29]. Cells from the matrix depicted all the characteristics of MSC phenotype and trilineage differentiation in appropriate culture conditions in our setup. The UC-MSCs further showed typical immunomodulatory properties as they very effectively suppressed the proliferation and activation of lymphocytes from peripheral blood as well as SF of RA patients in *ex vivo* conditions. Results from our study are in sync with previous reports regarding such ability of adipose tissue-derived or bone marrow-derived MSCs, which showed profound suppressive responses on Collagen II-reactive CD4 and CD8 lymphocytes from peripheral blood of patients with RA [30-32].

The conditioned medium obtained from the cultured human amniotic membrane-derived MSCs inhibited T cell proliferation [33] and differentiation of monocytes toward DCs and induced a shift toward an anti-inflammatory M2-like macrophages [34], indicating soluble factors released by MSCs also having similar properties. The UC-MSCs in our study showed the ability to tilt the delicate cytokine-balance from inflammatory to anti-inflammatory type as evident from the change in frequency and cytokine profile of Th1, Th2, Th17, and Treg cells. Wang *et al*. had earlier reported decreased expression of ROR-g, IL-17, and TNF-α in PBMCs from RA patients with severe and moderate disease activity on co-culturing with MSCs [35]. A similar study also reported that BM-MSCs decreased the percentage of cells producing IFN-γ, TNF-α, IL-17, and IL-2, in naive, effector, and memory T cell subsets from the peripheral blood of RA patients [36,37]. We also observed that the UC-MSCs functionally suppressed the CD8+ cytotoxic T-lymphocytes (CTLs) of SF and peripheral blood in terms of a decrease in percentage of granzyme B and perforin producing cytotoxic T-cells. Besides, these cells produced significantly reduced quantities of key pro-inflammatory cytokine – IFN-γ and IL-17, thereby indicating that the UC-MSCs would help in reducing tissue degeneration through functional down-modulation of CTLs, which may, in turn, alter the disease outcome by reducing the inflammation both systemically as well as locally at the site of injury.

The use of allogenic MSCs is further justified because the proliferation and clonogenic potential of endogenous stem cells in RA has been reported to be highly compromised. This defect was associated with decreased cellular telomere length and altered expression of genes implicated in focal adhesion and cell cycle pathways [30,31]. Our animal model study further demonstrated that the administration UC-MSCs reduced the clinical arthritis scores, synovial inflammation, and proliferation of osteoclasts with histopathological evidence of decreased cartilage and bone erosion in CIA rats. The therapeutic effects of MSCs were visible 2 weeks onwards post-treatment and were sustained till 6 weeks of observation without any further deterioration of arthritis parameters in CIA rats after a 1-time administration of UC-MSCs. Decreased antigen-specific Th1/Th17 cell expansion and induction of antigen-specific T-regulatory cells along with reduced incidence and severity of experimental arthritis were also reported by Bin Zhou *et al.*, using human adipose tissue-derived MSCs [38]. The role of osteoclast activation in systemic osteoporosis associated with inflammatory arthritis has been verified in various animal models. Inhibiting osteoclast differentiation and function become an important part of the treatment regimen when bone destruction is the therapeutic target. High expression of RANKL causes osteoclast differentiation in RA, while the OPG, a decoy receptor for RANK, will rescue the osteoclast differentiation. Our study shows, MSC-based interventions can help repair damaged joints in CIA rats without any adverse reaction in a sustained manner. We observed reduced expression of RANKL and increased expression of OPG in UC-MSC-treated CIA rats indicating a reversal of degenerative process in affected joints by MSC treatment. The findings of decreased bone erosion were further validated radiographically with the help of X-ray analysis of affected paws of MSC-treated CIA rats. A previous study conducted by Koichi *et al*. suggested that bone marrow-derived MSCs inhibit osteoclast differentiation *in-vitro* through constitutive production of OPG [39]. Another study by Ming *et al*. showed that the systemic delivery of TGF-β-transduced MSCs reduced the disease severity in terms of decreased clinical score, histological changes, and reduced production of IL-17, IL-21, and TNF-α and higher ratio of FoxP3+ cells to CD4 cells in the spleen and lymph nodes. *In-vitro*, the levels of the osteoclast-related markers TRAP, RANK, NF-ATc1, MMP-9, b3 integrin, and Cathepsin K mRNA decreased significantly following co-culture with TGF-β-transduced MSCs and *in-vivo* TGF-β-transduced MSCs decreased the osteoclast progenitor pool in the bone marrow [40]. Thus, UC-MSCs display marked anti-inflammatory and regenerative potential after transplantation in CIA rats, probably through paracrine mechanism: MSCs modulate the immune cells of both adaptive and innate immunity through secretion of soluble factors such as IDO, NO, PGE2, and IL-10 [41]. RANKL/OPG signaling plays an important role in synovial inflammation and joint destruction in RA. A study reported that changes in the RANKL/OPG system, involving an increase in RANKL and reduction in OPG in the peripheral blood and ST, represents an important mediator of bone resorption in RA-induced osteoporosis [42,43]. In our study, we found downregulation of factors related to bone destruction such as RANK, RANKL, TRAP, Cathepsin K, MMP3, MMP9, and NFATc1 after treatment with UC-MSCs, suggesting this to be a primary mechanism underlying the reduction of the bone and cartilage damage in the UC-MSC-treated CIA rats. Thus, our *in vitro* data, substantiated by the *in vivo* experiments in CIA rats, suggest that UC-MSCs can restore the tissue integrity through their “hit and go” mechanism leading to the systemic immunomodulation along with stimulation/mobilization of the endogenous repair mechanisms leading to disease attenuation.

## 5. Conclusion

Our findings support the therapeutic potential of UC-MSCs for autoimmune chronic inflammatory diseases such as RA through allotransplantation. This may be attributed to their dual ability to not only attenuate the exacerbated pathogenic immune activation seen in active arthritis patients but also facilitate the repair processes of the damaged tissues. *In-vitro* data highlight the role of UC-MSCs in potently decreasing the tissue injury in the synovial joint by suppressing the autoimmune effector T cell functions and at the same time reinstating the broken self-tolerance by inducing Treg proliferation and skewing the cytokine repertoire toward anti-inflammatory type. In addition, the *in-vivo* data reveal therapeutic effects of UC-MSCs in terms of slowing down the progression of disease activity and reversal of tissue damage processes along with triggering of tissue repair mechanisms. The most interesting observation is the sustainability of these effects for a longer duration of time. The data clearly indicate that the xenotransplantation of UC-MSCs in CIA rats leads to their interaction with the activated immune cells and down-modulate their destructive functions, besides contributing to the repair process by inducing the endogenous production of anti-osteoclastogenic and anti-inflammatory factors. Our findings add to the current perspective of using MSCs from an alternative source as a logical therapeutic approach for the treatment of arthritic joints.
